# A Promising Approach: Artificial Intelligence Applied to Small Intestinal Bacterial Overgrowth (SIBO) Diagnosis Using Cluster Analysis

**DOI:** 10.3390/diagnostics11081445

**Published:** 2021-08-10

**Authors:** Rong Hao, Lun Zhang, Jiashuang Liu, Yajun Liu, Jun Yi, Xiaowei Liu

**Affiliations:** 1 Department of Gastroenterology, Xiangya Hospital, Central South University, Changsha 410008, China; hr1205@csu.edu.cn (R.H.); pdsnj@126.com (J.L.); lyj19900801@126.com (Y.L.); 2 Hunan International Scientific and Technological Cooperation Base of Artificial Intelligence Computer Aided Diagnosis and Treatment for Digestive Disease, Changsha 410008, China; 3 Laboratory of Science and Technology on Integrated Logistics Support, National University of Defense Technology, Changsha 410072, China; zhanglun@nudt.edu.cn; 4 College of Intelligence Science and Technology, National University of Defense Technology, Changsha 410072, China

**Keywords:** SIBO, breath testing, cluster analysis, data driven, artificial intelligence

## Abstract

Small intestinal bacterial overgrowth (SIBO) is characterized by abnormal and excessive amounts of bacteria in the small intestine. Since symptoms and lab tests are non-specific, the diagnosis of SIBO is highly dependent on breath testing. There is a lack of a universally accepted cut-off point for breath testing to diagnose SIBO, and the dilemma of defining “SIBO patients” has made it more difficult to explore the gold standard for SIBO diagnosis. How to validate the gold standard for breath testing without defining “SIBO patients” has become an imperious demand in clinic. Breath-testing datasets from 1071 patients were collected from Xiangya Hospital in the past 3 years and analyzed with an artificial intelligence method using cluster analysis. K-means and DBSCAN algorithms were applied to the dataset after the clustering tendency was confirmed with Hopkins Statistic. Satisfying the clustering effect was evaluated with a Silhouette score, and patterns of each group were described. Advantages of artificial intelligence application in adaptive breath-testing diagnosis criteria with SIBO were discussed from the aspects of high dimensional analysis, and data-driven and regional specific dietary influence. This research work implied a promising application of artificial intelligence for SIBO diagnosis, which would benefit clinical practice and scientific research.

## 1. Introduction

Small intestinal bacterial overgrowth (SIBO) is an unbalanced status of intestinal micro flora characterized by an excessive concentration of bacteria in the small intestine, which presents with abdominal distention, nausea, diarrhea and other nonspecific symptoms in clinic [1,2,3]. Specific gastrointestinal diseases associated with SIBO include irritable bowel syndrome (IBS), inflammatory bowel disease and chronic pancreatitis, etc. SIBO is also closely related to extensive diseases such as type 2 diabetes, atherosclerosis, Bechet disease, autism spectrum disorder, chronic renal disease and other systemic diseases [2,4,5,6,7,8,9,10,11]. At the 21st Century Gut Microbiology Conference, it was proposed that the intestinal micro-ecosystem is a newly acknowledged physiology system that plays a significant part in human health. Either overgrowth or insufficient bacteria in the gut could result in various health issues. However, the misdiagnosis of SIBO is common in clinic with the lack of generally accepted diagnostic criteria. Thus, establishing better and well-acknowledged diagnostic criteria for SIBO is an urgent demand for both scientific research and clinical treatment.

For decades, several methods have been proposed, including small intestinal aspiration/culture, breath testing, clinical symptoms and diagnostic treatment [11,12]. Although not fully validated, small-bowel aspiration/culture is the current gold standard for SIBO diagnosis. Small-bowel aspiration is obtained during an upper gastrointestinal endoscopy by placing a sterile tube and collecting fluids in jejunum. Aspirates are then transferred immediately and cultured for bacteria growth. During the process of aspirates’ collection, contamination from oral and esophageal micro flora may lead to a false positive result and limited culture techniques could result in a false negative [13,14]. Moreover, the cut-off point of small-bowel aspiration is debatable with a historical view of bacterial concentration ≥10^5^ colony forming units (CFU)/mL and the current but not well-validated view of bacterial concentration ≥10^3^ CFU/mL [15,16,17,18,19,20]. The application of small-bowel aspiration is fairly limited in clinic, with other shortcomings including but not limited to invasive procedures, and time-consuming and complicated operations.

In clinical practice, breath testing is widely used for the diagnosis of SIBO with the benefits of being non-invasive, available and cost-friendly. H_2_ and CH_4_ are produced via bacteria in the gut by digesting carbohydrates, and are absorbed into blood circulation and finally expired through the lung [1]. Since human cells cannot produce H_2_ and CH_4_, when the amount of carbohydrates is settled the measurement of H_2_ and CH_4_ in exhaled breath could reflect the concentration of gut bacteria, which is the principle behind breath testing. A series of research has explored the sensitivity and specificity of breath testing. Breath testing based on lactulose presented with a sensitivity of 31–68% and a specificity of 44–100% [15]. In the North American Consensus (2017), it was suggested that until better data are available, an increase of ≥20 p.p.m. in hydrogen from baseline by 90 min should be considered a positive test for SIBO [21]. It was also noted in the consensus that there was a lack of a validated gold standard for diagnosing SIBO with breath testing and there was an urgent demand for a better-acknowledged diagnosis criterion.

With the absence of specific symptoms and limited application of small-intestinal aspiration in clinic, the diagnosis of SIBO is highly dependent on breath testing in clinical practice, which brings a question when exploring standard for breathing testing. Who are the SIBO-positive patients? In most situations, the diagnosis can be identified by pathological evidence or comprehensive indicators from symptoms, imaging and lab testing, but SIBO is a nonspecific disease identified mostly based on breath testing, which leads to the dilemma of identifying positive controls. For decades, most research focusing on breath testing took “small-bowel aspiration positive patients” or “patients with abdominal discomfort” as “SIBO patients” and clarified the diagnosis criteria with a cross-over trail. However, as we discussed above, patients with abdominal discomfort cannot represent SIBO patients. The high risk of false positive and debatable cut-off of small-bowel aspiration resulted in aspiration positive patients as suspectable SIBO-positive controls. Thus, how to explore the diagnosis criteria for SIBO without definitive SIBO patients remains an urgent challenge for clinical and scientific purposes.

In recent decades, artificial intelligence (AI) developed rapidly and was applied dramatically in medicine. Deep learning, reinforcement learning, transfer learning, data mining and other AI algorithms are widely used in various research and applications that used to be considered achievable only by humans, such as autonomous diagnosis, drug development and image interpretation [22,23,24,25,26]. Esteva reported an AI system for skin-cancer classification using deep neural networks with datasets containing 129,450 clinical images of 2032 different diseases [27]. A comparison between experienced dermatologists and the AI system presented no significant difference in terms of classifying skin cancers. Yishan He summarized an AI-based detection and diagnosis tool for gastrointestinal lesions, which demonstrated that AI was promising in providing an effective and practical method for lesion detection and characterization with endoscopy [28].

Machine learning is the core of artificial intelligence (AI). It specializes in computer simulation or implementing human learning behaviors to acquire new skills and reorganize existing knowledge structures to continuously improve its performance. According to the training data, machine learning can be divided into supervised learning and unsupervised learning. For a given group of data (***X***, ***Y***), in which ***X*** is the data example and ***Y*** is the corresponding label, machine learning provides a series of methods to build up models that could best fit the data. Supervised learning methods such as K-Nearest Neighbor (KNN) and Support Vector Machine (SVM) could be applied when ***Y*** is available. However, when ***Y*** is not provided, in which situation the supervised learning method does not work anymore, unsupervised learning should be considered. 

Cluster analysis is a sub-branch of unsupervised machine learning that attempts to fit the training data without any prior knowledge of the classes. Cluster analysis is a hot topic in AI when dealing with unlabeled data [29,30]. It is also helpful to build up a classification model when no prior knowledge of the data is available [31,32]. Data are divided into several clusters according to the inherent nature and regularity of the data. With the proverb which says “birds of a feather flock together”, the goal of clustering is to make the datasets in the same cluster share high similarity while datasets in different clusters present with distinction. Application of cluster analysis on medical data processing has drawn much attention in recent years [33,34,35,36,37,38]. Marks-Garber presented a cluster analysis-based clinical profiling of Idiopathic Pulmonary Fibrosis (IPF), which may help in developing a diagnostic algorithm for earlier diagnosis of IPF [39]. Manuel Rubio-Rivas investigated clinical phenotypes and prediction of chronicity in sarcoidosis using cluster analysis in a cohort of 694 patients [40]. He identified 6 different clinical patterns with similar phenotypic variables and predicted chronicity, which may be helpful in improving the efficacy of clinical decisions.

Could we break through the dilemma of identifying SIBO patients with the help of AI techniques? Past clinical research focused on breath-testing diagnosis criteria for SIBO, most based on the idea of classification, with which “positive” and “negative” are labeled and an analytical model is built based on the labeled data. When new data come, the model would classify it into the positive group or negative group. However, classification is not the best way for SIBO since there is no identified positive SIBO patient. Can we analyze SIBO with the idea of clustering instead? With clustering, there are no previously labeled data. Data are gathered into groups automatically with inner characteristics, in which process there are no human biases. 

There is a lack of a universally accepted cut-off point for breath-test diagnosis of SIBO, and the dilemma of defining “SIBO patients” has made it more difficult to explore the gold standard for a breath test. How to validate the gold standard for a breath test without defining “SIBO patients” has become an imperious demand in clinic. This project was proposed to use cluster analysis to process breath-testing data collected from Xiangya Hospital and develop a new diagnosis standard for SIBO by identifying new patterns of hydrogen generation.

## 2. Materials and Methods

### 2.1. Subjects

Data samples from 1101 lactulose-based breath tests were collected in the past 3 years at Xiangya Hospital, Central South University. We excluded 8 data samples with atypical interval time, and 22 were excluded because of incomplete process. There were 22 testing results presented with all zeros in 8 time points, which could be the result of carelessness in equipment operations. The source code raised a reading error when opening and loading data from some data files, which were damaged somehow. We tested 8 data samples fewer than 8 times during a breath test; typically, 6 or 7 bags of breath air were collected and tested. The 1071 breath-testing samples were applied in this study. The research was approved by the Ethics Committee of Xiangya Hospital, Central South University (identification code: 202004283, identification date: 10 April 2020). Written informed consent was obtained from each patient enrolled.

### 2.2. Breath Testing

Patient preparation: Patients were instructed to avoid dairy products, soy products and high-fiber vegetables, which can produce H_2_. Rice, meat and eggs are a suitable source of food and over satisfaction should be avoided. Patients were encouraged to eat rice soup the evening before testing and start fasting at least 12 h before the test. Soft drinks, gum and smoking (including passive smoking) were to be avoided. Patients were not allowed to eat, drink, sleep and exercise during the test.

Gas collection: Fast gas was collected in the first bag by installing “specimen bag 1” on the breathing tube. Patients were asked to hold the filter in their mouth and exhale calmly (avoid deep inhalation and deep exhalation). After blowing up the specimen bag, the bag was taken off immediately. After the first exhalation, 10 g of lactulose was mixed in 250 mL water, and patients were asked to drink it within 30 s. Gas was collected every 20 min as described before, until finishing the 8th bag in 140 min. Then, 8 bags of gas collection were tested in the gastroenterology laboratory of Xiangya Hospital within 24 h. 

Gas testing: After turning on the detection instrument and running it for a few minutes to exhaust the residual gas in the machine, we drew 20 mL of “standard gas” into the breath-tracker gas chromatograph (Quintron Instrument Co.inc, Milwaukee, WI, USA) through the filter tube for calibration. After calibration, we used a syringe to extract the gas in the 20 mL sample bag and inject it into the machine through the filter hall for measurement. 

### 2.3. Cluster Analysis

Focusing on the research interest of this paper, breath-testing data with a test interval of 20 min were collected for machine learning, and an optimized diagnosis criterion without subjective biases was expected.

#### 2.3.1. Clustering Tendency Evaluation

For a given dataset, clustering-tendency evaluation is necessary before clustering since analysis is only meaningful when there is nonrandom structure in the data. Clustering-tendency evaluation determines whether a given dataset has a nonrandom structure that can lead to meaningful clustering. When there is no nonrandom structure in a dataset, such as uniformly distributed points in the data space, clusters for this dataset could still be calculated with a clustering algorithm, but the clusters are random and meaningless. Clustering requires nonuniform distribution of data.

Hopkins Statistic is used to verify the randomness of spatially distributed variables. It can be applied to evaluate the clustering tendency of a dataset. Hopkins Statistic is calculated as the following: Let ***X*** be the dataset for cluster analysis, which contains *n* samples. First, generate a dataset ***S*** that contains *r* (*r* < *n*) samples randomly selected from ***X*** and let *α*_1_, *α*_2_, …, *α_r_* be the distance of the sample in ***S*** to their nearest neighbors within the original dataset ***X***. Secondly, generate a synthetic dataset ***R*** randomly in the domain of the data space and let *β*_1_, *β*_2_, …, *β_r_* be the distance of the sample in ***R*** to their nearest neighbors within the original dataset ***X***. Then, the Hopkins Statistic *H* can be evaluated using the function

(1) $$H=\frac{\sum_{i=1}^{r}\beta_{i}}{\sum_{i=1}^{r}\alpha_{i}+\sum_{i=1}^{r}\beta_{i}}=\frac{\sum_{i=1}^{r}\beta_{i}}{\sum_{i=1}^{r}\left(\alpha_{i}+\beta_{i}\right)},$$

Theoretically, the value of the Hopkins Statistic varies from 0 to 1 for different datasets. A uniformly distributed dataset will have a Hopkins Statistic value of 0.5 since the value of *α_i_* and *β*_ι_ are very similar. For a clustered data, the Hopkins Statistic will be closer to 1 since the value of *α_i_* is much lower than *β_i_*. Therefore, a high value of the Hopkins Statistic *H* indicates a high tendency of data points [41]. Practically, it can be believed that the dataset has high clustering tendency if the Hopkins Statistic is in the range (0.7, 1). The Hopkins Statistic is useful to evaluate the dataset before clustering. However, there is no ability to reveal cluster numbers within the dataset.

#### 2.3.2. K-means Cluster Algorithm

K-means is a typical unsupervised learning method for cluster analysis. The core idea of the K-means clustering algorithm is to divide the data objects into different clusters according to their similarity, so that the generated clusters are as compact and independent as possible.

Similarity is usually measured by space distance between two data examples. The smaller the distance, the higher the similarity. There are three imperative properties of distance:

1. non-negativity, *d*(*i*, *j*) > 0 if *i* ≠ *j*, and *d*(*i*, *i*) = 0
2. symmetry, *d*(*i*, *j*) = *d*(*j*, *i*)
3. triangle inequality, *d*(*i*, *j*)<= *d*(*i, k*) + *d*(*k*, *j*)

The general formula for distance calculation is the Minkowski Distance, which is

(2) $$d(i,j)=\sqrt[{h}]{{\left|x_{i1}-x_{j1}\right|}^{h}+{\left|x_{i2}-x_{j2}\right|}^{h}+\cdots +{\left|x_{in}-x_{jn}\right|}^{h}},$$

where *n* is dimension of data example. When *h* = 1, the distance will be Manhattan Distance. When *h* = 2, the distance will be Euclidean Distance, which is most widely used. In this paper, Euclidean Distance is used unless otherwise specified.

Without losing generality, assuming the training dataset is $D=\{X_{1},X_{2},\cdots ,X_{n}\}$, centers of *k* clusters is $\mu =\{\mu_{1},\mu_{2},\cdots ,\mu_{k}\}$, and sample number of each cluster is $N=\{N_{1},N_{2},\cdots ,N_{k}\}$, if the dataset is well clustered, for any sample in cluster *j*, the distance to *μ_j_* will be less than any other cluster centers.

Construct a target function as the following,

(3) $$J(\mu )=\frac{1}{2}\sum_{j=1}^{k}\sum_{i=1}^{N_{j}}d^{2}(X_{i},\mu_{j}),$$

thus, for a clustering algorithm, the dataset will be well clustered only if *J*(*μ*) is equal to its minimal value. According to the idea of convex optimization, partial derivatives function (3) with respect to *μ_j_*, and set derivatives equals to 0,

(4) $$\frac{\partial J(\mu )}{\partial u_{j}}=-\sum_{i=1}^{N_{j}}d(X_{i},u_{j})=0,$$

the following function can be derived by solving function (4),

(5) $$\mu_{j}=\frac{1}{N_{j}}\sum_{i=1}^{N_{j}}X_{i},$$

When applying the K-means cluster algorithm, first, *k* samples are randomly selected as the center of the initial *k* clusters, and then the remaining objects are assigned to the nearest cluster according to their distance from the center of mass of each cluster. The iterative relocation process is repeated until the objective function is minimized to find the center of mass of the newly formed cluster.

The K-means cluster algorithm is relatively efficient compared to other cluster algorithms. When dealing with large datasets, the algorithm can also guarantee good scalability. However, there are also some cautions when using K-means. First, the number of clusters *k* must be predefined before clustering. Secondly, the K-means algorithm is not good at dealing with non-convex-shaped clusters. Finally, the algorithm usually terminates at local optimum, but it can be improved by a global optimization technique.

#### 2.3.3. DBSCAN Cluster Algorithm

DBSCAN is another widely used cluster analysis method in the data-mining area. Unlike K-means, which is a distance-based method, DBSCAN is a density-based cluster method. Clusters were identified as high-density areas that were separated by low-density areas in DBSCAN. It is able to discover oddly shaped clusters and does not require a predetermined cluster number. Additionally, DBSCAN has advantages in processing data with noises.

For a dataset $D=\{X_{1},X_{2},\cdots ,X_{n}\}$, assuming x∈D, then ε neighbor of x can be defined as

(6) $$N_{\varepsilon }(x)=\{y\in D:d(x,y)\leq \varepsilon \},$$

Apparently, $x\in N_{\varepsilon }(x)$. Then the density of dataset ***D*** at sample x, ρ(x), is defined as the number of samples in the ε neighbor of x. 

A sample x is a core point in dataset ***D*** when ρ(x)>M, where M is a nonnegative integer. And the set of all core points within dataset ***D*** is donated as ***D***_c_.

For any x,y∈D, *y* is directly density-reachable from *x* if $x\in D_{c},y\in N_{\varepsilon }(x)$. Moreover, for any x,y∈D, if there exists a sample sequence *p*_1_, *p*_2_, …, *p*_T_, where *p*_1_ = *x*, *p*_T_ = *y*, and *p_i+_*_1_ is directly density-reachable from *p_i_*, then *y* is density-reachable from *x*.

DBSCAN regards the maximum density reachable set derived from the density reachable relationship as a cluster. When applying DBSCAN method, radius of ε neighbor and minimum sample number of core point should be specified. Then the algorithm starts from a randomly selected core point, finds out all density-reachable samples and denotes them as a cluster. After this, a new core point that belongs to no previous discovered clusters is selected to find a new cluster by searching all density-reachable samples. Such procedures continue until all core points are checked.

Compared with K-means algorithm, the difference between them is that no predetermined number was needed as a vital parameter in DBSCAN. Moreover, DBSCAN can find the clustering cluster of any shape, rather than being generally only used for convex sample clustering classes such as K-means.

#### 2.3.4. Clustering Validation

When conducting cluster analysis, the cluster algorithm returns a model that divides *n* samples into *k* clusters. However, the algorithm never promises a meaningful clustering result. It is vital to validate quality of cluster analysis results to guarantee further analysis and application. A criterion for clustering validation is necessary after applying the cluster algorithm. 

Silhouette score is a usually used evaluation method of clustering effect. It combines two factors, namely cohesion and separation. It can be used to evaluate the influence of different algorithms or different parameters on clustering results based on the same original data. Silhouette score is calculated as the following:

1. For sample *X_i_*, calculate its average distance to samples within the same cluster, let the average distance be *a_i;_*
2. For sample *X_i_*, calculate its average distance to samples of any clusters without *X_i_*, let the average distance be *b_i;_*
3. The Silhouette score of *X_i_* can then be calculated by the formula

(7) $$S_{i}=\frac{b_{i}-a_{i}}{\max(a_{i},b_{i})}$$

4. The overall Silhouette score is the average of all data points.

Theoretical value of Silhouette score is between −1 and 1. The higher the Silhouette score is, the better the clustering is. The absolute values of Silhouette score provide a good intuitive evidence of the clustering quality.

### 2.4. Data Processing Procedure

**Figure 1:**
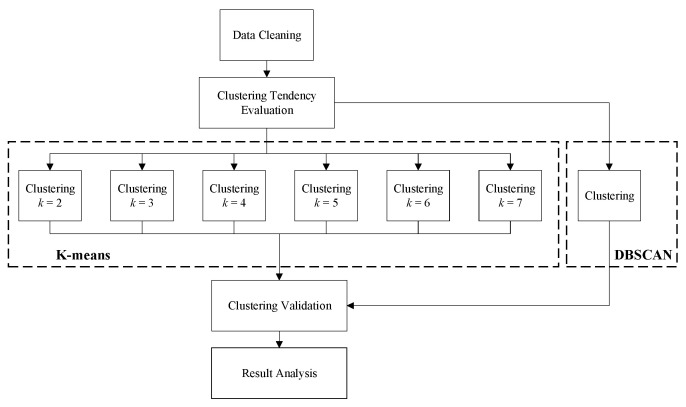
Data processing flow-chart.

Shown in Figure 1 is the data processing flow-chart in this paper, which mainly consists of 5 steps.

First, data cleaning is carried out after breath-testing data collection. Data exclusion strategy was described in detail in Section 2.1.

Secondly, Hopkins Statistic of the dataset is evaluated for confidence of cluster analysis.

Thirdly, K-means cluster analysis is conducted with different cluster numbers, namely *k* = 2, 3, 4, 5, 6, and 7. DBSCAN method is applied to analyze the data as well.

Then, Silhouette scores corresponding to each cluster number are evaluated to decide the best cluster result for further analysis.

Finally, the best results of both K-means and DBSCAN clustering are analyzed and discussed.

## 3. Results

### 3.1. Data Visualization

Among the dataset, 1071 samples qualified for further analysis. Before further analysis, the baseline of breath test H_2_ level was subtracted from test values.

The datasets ready for analysis were saved in an 8 × 1071 matrix. Every column of the matrix corresponded to a data sample. Numeric value in each row of a specific data sample was H_2_ change value relative to the baseline.

**Figure 2:**
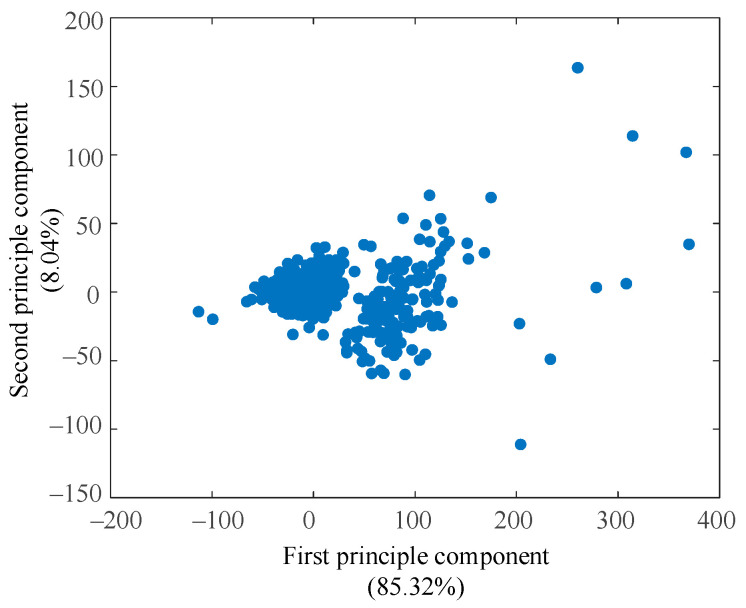
Data visualization using PCA.

A principal component analysis (PCA) was conducted for an intuitive understanding of the dataset. Figure 2 illustrated data distribution of the first two principal components, which contained 93.96% of data variability (85.32% and 8.04%, respectively). The third component contained only 2.43% of variability, which could be ignored in visualization.

Data distribution in Figure 2 highly suggested data aggregation. The most compact data group gathered around (0, 0), among which data point markers covered up each other. Another group of data lay next to the first group, but it was relatively loose with clear gaps observed between data points. Several data points between the two groups of data were ambiguous to be classified into the first or second group from intuition. In addition, some sparse and sporadic data points spread at the right side of the figure. The outline shape of the visualized data groups exhibited spherical distributions, which indicated that the K-means clustering algorithm was suitable for our dataset.

### 3.2. Clustering Tendency

After data cleaning and visualization, clustering tendency evaluation was demonstrated with the Hopkins Statistic of dataset calculation. The Hopkins Statistic of the dataset was 0.9460, which was very close to 1. The Hopkins Statistic of the dataset highly suggested that the dataset has strong clustering tendency.

### 3.3. K-Means Cluster Results 

When clustering with K-means, the number of cluster *k* was an important input parameter to divide the dataset into clusters. Centroids of each cluster were randomly initialed without any possible subjective biases. The K-means algorithm was run with *k* = 2, 3, 4, 5, 6, and 7. The initial value *k* = 2 was selected classically since the North American criterion determines 2 kinds of SIBO status: positive or negative. The last evaluated value *k* = 7 was selected based on expert knowledge.

**Figure 3:**
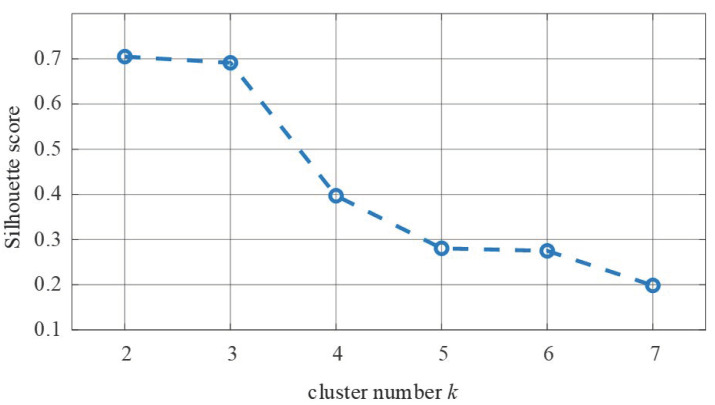
Silhouette score trend with respect to cluster numbers.

The Silhouette score was used to validate the clustering result. The Silhouette score corresponded to different cluster number and was calculated after clustering using the K-means algorithm. Results are listed in Table 1. Figure 3 shows the tendency of the Silhouette score with respect to the cluster number.

Table 1 and Figure 3 indicate that the Silhouette score decreased as the cluster number increased from 2 to 7. When the cluster number changed from 3 to 4, the Silhouette score decreased dramatically to almost half of the previous value. If only the best Silhouette score was considered, then *k* = 2. However, it was unignorable that the Silhouette score was almost the same when *k* = 2 or 3; the difference was less than 5%. Therefore, the cluster results of *k* = 2 and *k* = 3 were both illustrated and analyzed.

**Figure 4:**
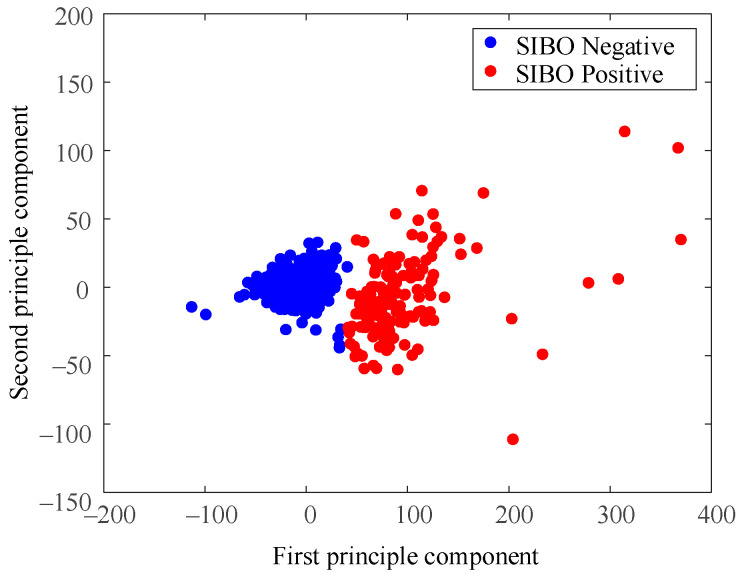
Cluster result when *k* = 2.

Figure 4 shows data distribution of clustering results when *k* = 2.

The most compact data group and some scattered data points around it were regarded as a cluster (blue ones in Figure 4) while the relatively loose data group and spread data points at the right side were regarded as the other cluster (red ones). The 3 blue data points located at the lower right corner of the first cluster were noticeable since they seem closer to the second cluster(red). The demarcation line between the two clusters was not very clear. 

**Figure 5:**
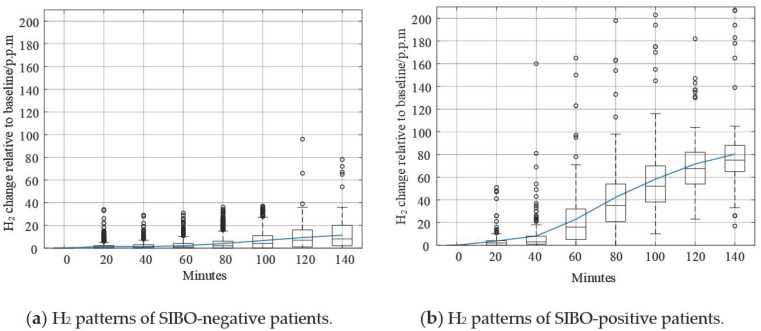
Graphical demonstration of the boxplot and mean values of clusters when *k* = 2.

Different typologies of data curves are shown in Figure 5. The solid curve in the figure was mean value of H_2_. In Figure 5a, the H_2_ curve is approximately flat since the increase of H_2_ level was not remarkable. Additionally, the mean value and median value of H_2_ level did not exceed 20 p.p.m. within 140 min. According to the North American consensus, this pattern could be confidently regarded as SIBO. However, it was also noticeable that some data samples of this kind exceed 20 p.p.m. within 90 min, which challenged the North American consensus. In Figure 5b, the H_2_ level mean value curve increased steadily after taking lactulose and exceeded 20 p.p.m. at 60 min. This curve could be confidently believed as SIBO-positive.

**Figure 6:**
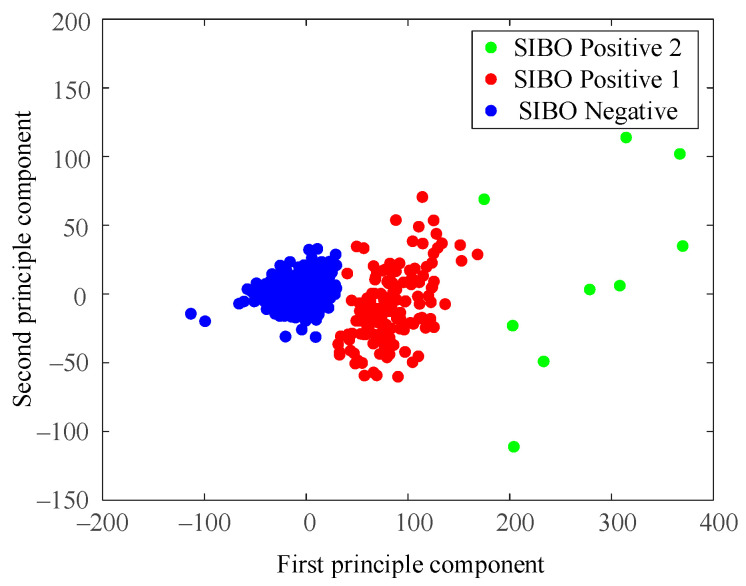
Cluster result when *k* = 3.

Figure 6 shows data distribution of the clustering result when *k* = 3. A major difference with the result of *k* = 2 was that the spread of sparse data points at the right side were regarded as a new cluster. The K-means algorithm treated those data points as a cluster different from the two relatively compact ones.

**Figure 7:**
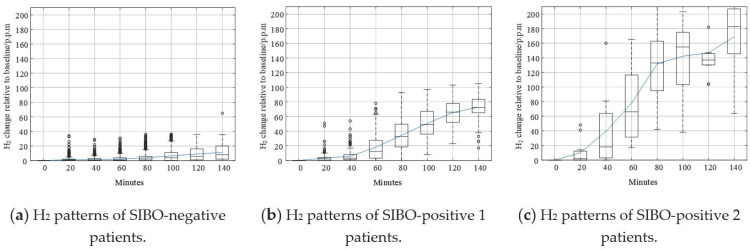
Graphical demonstration of the boxplot and mean values of clusters when *k* = 3.

Shown in Figure 7 are different typologies of data curves. The solid curve represents the mean value of H_2_. Like that in Figure 5a, the H_2_ curve in Figure 7a is relatively flat compared to other curves. The increase of H_2_ level was not remarkable, and the mean value and median value of H_2_ did not exceed 20 p.p.m. within 140 min. According to the North American consensus, this pattern could be confidently regarded as SIBO-negative since less H_2_ gas was produced after taking in lactulose. In Figure 7b, the H_2_ curve increased steadily after the beginning of breath testing. The H_2_ increasing level exceeded 20 p.p.m. around 60 min. The trend of the pattern in Figure 7b is similar to the pattern in Figure 5b. In Figure 7c, the H_2_ curve increased dramatically after the beginning of the breath test. The H_2_ increasing level exceeded 20 p.p.m. earlier than 60 min. The increasing speed slowed down after 80 min, but kept increasing until the end of breath testing.

Though the Silhouette score of *k* = 2 is larger than that of *k* = 3, there were more outliers in Figure 5 than Figure 7. In Figure 5, the outliers deviated further from the maximum of box plots as well. These phenomena suggest that *k* = 3 was better than *k* = 2. 

**Figure 8:**
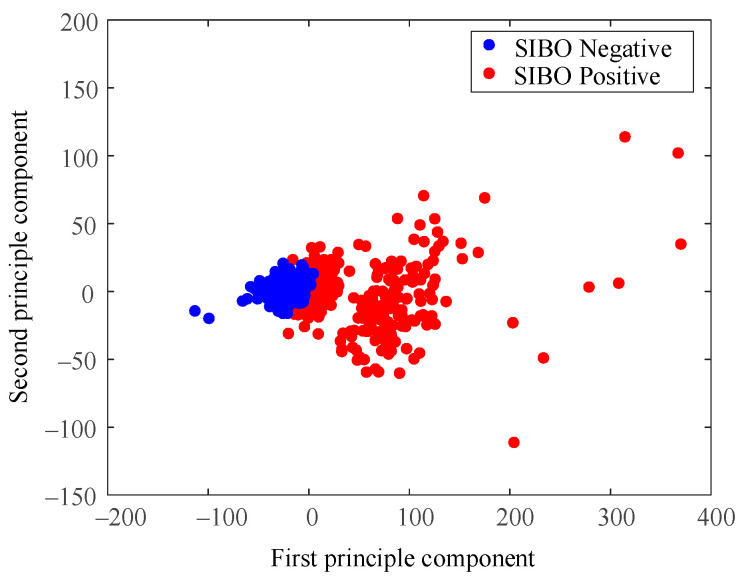
Diagnosis results according to the North American Consensus.

Figure 8 is the result of using the North American Consensus presented with PCA dimensional reduction. Samples were labeled SIBO-negative in blue if the H_2_ level increased less than 20 p.p.m. from baseline within 90 min with the standard of 2017 North American Consensus. The North American Consensus threshold was stiff since it seemed to classify the samples into positive group and negative group by forcing a cut-off line in the data space without considering the data boundaries. As a result, a large amount of data samples were regarded as SIBO-positive by the North American Consensus, though they were very similar to the negative ones from the perspective of data-space distribution.

### 3.4. DBSCAN Cluster Results

**Figure 9:**
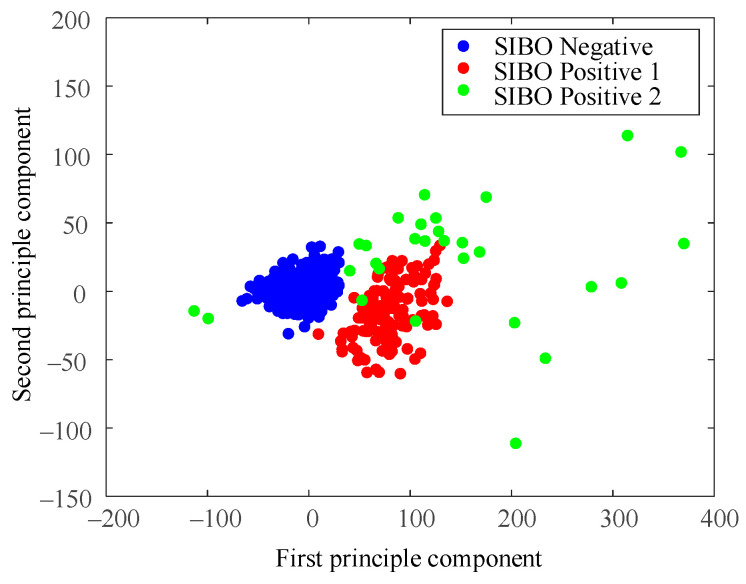
Cluster result using DBSCAN.

The cleaned data were analyzed using the DBSCAN algorithm as well. Shown in Figure 9 is data distribution of clustering results using the DBSCAN method. The cluster result of DBSCAN is similar to that of K-means when *k* = 3. The major difference was the relative low-density data points around high-density data points. To be more specific, the group of green points above red ones, and the two green points on the left of blue ones. They were clustered into different classes when using the DBSCAN method. Such results were caused by the difference between the two methods in basic theory.

**Figure 10:**
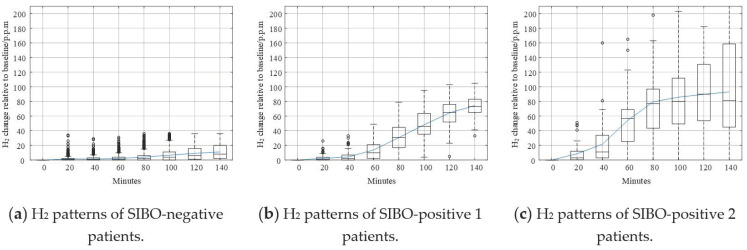
Graphical demonstration of the boxplot and mean values of clusters using DBSCAN.

Figure 10 shows different breath data curves corresponding to every class identified by the DBSCAN method. Like the curves in Figure 7, the solid curve represents the mean value of H_2_. The H_2_ curve in Figure 10a could be confidently regarded as SIBO-negative since the mean value and median value of H_2_ did not exceed 20 p.p.m. within 140 min. Figure 10b,c are both SIBO-positive, but they were identified as different SIBO-positive types by DBSCAN according to their density in data space.

## 4. Discussion

SIBO is a disease identified with small intestinal bacteria overgrowth and it presents with abdominal distention, diarrhea or even developmental retardation when nutrition absorption is blocked. SIBO shares an extensive relationship with overall health, but the diagnosis of SIBO is debatable in clinic with the lack of a well-acknowledged diagnosis standard. Breath testing is the most significant and widely applied method for SIBO diagnosis. However, the criterion of breath testing in diagnosing SIBO remains invalidated. The difficulty of exploring SIBO diagnosis with breath testing is identifying SIBO-positive patients when the diagnosis of SIBO is based mainly on breath testing. Past research solved this problem with two ways: defining SIBO-positive with either small intestinal aspiration/culture positive or patients with unspecific symptoms. However, neither of these two could optimally represent SIBO patients as we discussed in introduction. In our research, we proposed a solution for this dilemma with cluster analysis, in which datasets were gathered with their internal characteristics and information without human biases.

During the research, both K-means and DBSCAN clustering methods were used to analyze the data. We confirmed the clustering tendency of the breath testing dataset by calculating with Hopkins Statistic. The Hopkins Statistic of the 1071 samples collected from Xiangya Hospital for the past 3 years highly suggested that the dataset presented with a strong clustering tendency and clustering analysis was suitable for breath testing. As a classical method of cluster analysis, K-means clustering was applied and a satisfying clustering effect was evaluated with a Silhouette score. DBSCAN was applied to the dataset as another classical clustering method as well. 

As shown in Figure 4 and Figure 6, the dataset was clustered to 2 and 3 groups with the intrinsic information. Both of these two clustering strategies presented with good clustering effect as the demarcation line between the groups was clear. There was a difference when the dataset was clustered into different number of groups. As shown in Figure 4 when the dataset was clustered into 2 groups, the 3 blue points located at the lower right corner of the first cluster (SIBO-negative) were noticeable since they seem closer to the second cluster (SIBO-positive). However, when the dataset was clustered into 3 groups, the 3 points that were considered to be first cluster were now assigned into second cluster, which seems intuitively more reasonable. The spread of sparse data points at the right side were regarded as a new cluster, which is different from the two relatively compact ones in Figure 6. This result suggested that clustering the dataset into 3 groups (*k* = 3) seemed to be more reasonable. The patterns of each group are also illustrated in Figure 5 and Figure 7. More wild data points were observed in Figure 5, which indicated that there was a potential risk of misclassification. The patterns in Figure 7b,c were significantly different from each other, which indicated that clustering of 3 groups might be a more appropriate choice. 

When comparing Figure 6 and Figure 9, the major difference between K-means and DBSCAN analysis results was the SIBO-positive patterns. K-means regarded the spread sparse data points on the right side as the third cluster, while DBSCAN regarded the relative low-density data points spread around the relative high-density data groups as the third cluster. Comparing Figure 7 and Figure 10, it could be easily observed that H_2_ curves share almost the same patterns in (a) and (b). Differences mainly appeared in (c). In Figure 7c, the H_2_ curve rose dramatically to more than 120 p.p.m. within 80 min and then steadily increased to more than 160 p.p.m. in the following 1 h. In Figure 10c, the H_2_ curve also rose dramatically within 80 min and then increased slowly but steadily. Both K-means and DBSCAN effectively identified several significant clusters with the dataset, which supported our hypothesis of applying a clustering algorithm to SIBO diagnosis.

The overall results based on cluster analysis were in accordance with the standard in the 2017 North American Consensus, while they also presented with differences. For SIBO-negative, the mean value and median value of the H_2_ level increase relative to baseline was less than 20 p.p.m. within 90 min, which was in agreement with the Consensus. However, as shown in Figure 8, there were samples that were diagnosed positive with the 2017 North American Consensus clustered to the negative group, which indicated that some data samples that were believed SIBO-positive according to North American Consensus standard should be regarded as SIBO-negative according to cluster analysis since they share a higher similarity to SIBO-negative samples in the data space. The North American Consensus threshold was stiff since it seemed to classify the samples into a positive group and a negative group by forcing a cut-off line in the data space without considering the data boundaries. As a result, a large amount of data samples was regarded as SIBO-positive by consensus though they shared considerable similarity to the negative ones from the perspective of data-space distribution. A considerable numbers of patients who were diagnosed positive by the consensus should be SIBO-negative according to the clustering analysis. 

In the view of data analysis, diagnosis of SIBO using North American Consensus is simply a threshold comparison. A threshold for diagnosis could be easily carried out in clinical practice based on numerical examinations such as blood tests. However, breath-testing data are more like a vector in 8-dimensional space, in which each dimension corresponds to an H_2_ value at a time point. Unlike in the North American Consensus, which compares data from 2 time points (0 min and 90 min), more information is enrolled in the analysis based on the machine-learning algorithm. The similarity of two data samples is evaluated based on their density or distance in data space. The relative difference in value at each time interval is counted as well. Additionally, high-dimensional analysis provides the potential to precisely classify SIBO-positive samples into several patterns. A threshold diagnosis using the North American Consensus could only distinguish SIBO-positive and -negative with the restriction by its intrinsic nature. Cluster analysis is a data-driven method. No prior knowledge or background information is needed for unsupervised learning methods, which means they can “learn” the intrinsic nature and underlying knowledge that are veiled deep within the messy and human-unfriendly data. With the dilemma of identifying SIBO-positive patients in clinic, a cluster analysis seems to be a perfect choice for SIBO diagnosis. Moreover, as the hospital accumulates clinical test data day by day, a data-driven method is able to automatically update the model or renew the algorithm by learning from the newly generated data. 

Meanwhile, the regional-specific dietary structure may influence the collected dataset and contribute to the difference in the SIBO diagnosis standard from Consensus and our result. Unlike in the North American Consensus, which used data collected mostly from North American and Europe, data from Xiangya Hospital were collected from Hunan Province, South China. The result of breath testing is influenced by multiple factors including PPI (proton pump inhibitor), antibiotic usage history, position, sports activities and dietary structure [13,42,43,44]. A harder found high-caloric diet could result in significant gas retention [45,46]. Multiple studies demonstrated that a diet with abundant beans, potato, flour and corn led to increased H_2_, while meat as well as rice would not. Numerous studies showed dietary structure could impact bacteria flora in multiple ways [46]. For example, in the Chinese dietary structure, the energy supply from fat is higher than that in Europe, while dietary fiber and vitamins are lower than in Europe and Japan [47]. With the evidence of food structure influencing gut bacteria that we posed in the introduction, we considered the question of whether the breath-testing baseline and pattern vary in different areas with different dietary structures. In the North American Consensus (2017), an increase of ≥20 p.p.m. from baseline in hydrogen by 90 min should be considered a positive test for SIBO and a decrease of ≥10 p.p.m. means methane positivity, based on a database mostly from North American and European areas [21]. With the experience from Xiangya Hospital, we found that CH_4_ rising >15 p.p.m. related to clinical symptoms with the background of spicy food flavors in Human Province. Until now, there was no research about the region-specific breath-testing baseline based on various dietary structures in different areas. In future work, we plan to further explore the region-specific diagnosis standard in different areas based on the influence of dietary structure.

Our results showed the advantages of artificial-intelligence application in adaptive breath-testing diagnosis criteria with SIBO. The method of data clustering presented with natural advantages with data which own a strong clustering tendency. Our research was based on breath-testing data collected from real clinical work without any prior knowledge or expert experience. The patterns we found were relying on objective, automated and intelligent analysis, which could reflect internal characteristics of gut bacteria. Furthermore, comparing with the standard based on single-point threshold in the North American Consensus, our method analyzed data distribution in high-dimensional space, in which much more information was included. There were also challenges and limitations in our work. There was potential risk of deviation in sample collection. Doctors would prescribe breath testing to patients only when they were suspicious of SIBO, and thereby there was a lack of data from people presented without any symptoms. This problem could be solved by enrolling healthy volunteers or conducting regular non-discriminatory tests on patients in the future work.

## 5. Conclusions

A new SIBO diagnosis criterion was proposed in this paper based on cluster analysis using K-means and DBSCAN algorithms. Breath-testing datasets from 1071 patients were collected from Xiangya Hospital from the past 3 years. This research work implied the potential of applying machine learning techniques to clinical datasets for SIBO diagnosis. Advantages of artificial intelligence application in adaptive breath testing diagnosis criteria with SIBO were discussed from the aspects of high dimensional analysis, data-driven and regional-specific dietary influence. This research work also developed a promising diagnosis standard for SIBO, which would benefit clinical practice and scientific research.

## Figures and Tables

**Table 1:** Silhouette scores of different cluster numbers.

*k*	2	3	4	5	6	7
Silhouette score	0.7050	0.6913	0.3971	0.2807	0.2751	0.1986

## Data Availability

The data presented in this study are available on request from the corresponding author. The data are not publicly available due to patients’ privacy.
